# ViSimpl: Multi-View Visual Analysis of Brain Simulation Data

**DOI:** 10.3389/fninf.2016.00044

**Published:** 2016-10-07

**Authors:** Sergio E. Galindo, Pablo Toharia, Oscar D. Robles, Luis Pastor

**Affiliations:** ^1^Universidad Rey Juan Carlos, Madrid, Spain; ^2^Universidad Politécnica de Madrid, Madrid, Spain; ^3^Center for Computational Simulation, Universidad Politécnica de Madrid, Madrid, Spain

**Keywords:** brain simulation, spiking neurons, visualization software, multi-view analysis, spatial and temporal visualization

## Abstract

After decades of independent morphological and functional brain research, a key point in neuroscience nowadays is to understand the combined relationships between the structure of the brain and its components and their dynamics on multiple scales, ranging from circuits of neurons at micro or mesoscale to brain regions at macroscale. With such a goal in mind, there is a vast amount of research focusing on modeling and simulating activity within neuronal structures, and these simulations generate large and complex datasets which have to be analyzed in order to gain the desired insight. In such context, this paper presents ViSimpl, which integrates a set of visualization and interaction tools that provide a semantic view of brain data with the aim of improving its analysis procedures. ViSimpl provides 3D particle-based rendering that allows visualizing simulation data with their associated spatial and temporal information, enhancing the knowledge extraction process. It also provides abstract representations of the time-varying magnitudes supporting different data aggregation and disaggregation operations and giving also focus and context clues. In addition, ViSimpl tools provide synchronized playback control of the simulation being analyzed. Finally, ViSimpl allows performing selection and filtering operations relying on an application called NeuroScheme. All these views are loosely coupled and can be used independently, but they can also work together as linked views, both in centralized and distributed computing environments, enhancing the data exploration and analysis procedures.

## 1 Introduction

Human interest in understanding our brain can be traced back to the very beginning of science or even of mankind. There are two reasons for this interest: first, our brain is what makes us *human* and, second, many of the diseases or accidents affecting our brain have very strong personal costs, regarding disability and quality of life [in addition to economic costs (Olesen et al., 2012)].

Progress in brain research has been impaired mainly by the brain’s incredible degree of complexity and by the unavailability of adequate tools for its study. Regarding complexity, with around 10^11^ neurons and 10^15^ synapses (in addition to glia and vasculature, with influence also in brain function), the brain is the most complex dynamic network under study (Sporns et al., 2005). Regarding analysis tools, historically, there have not been too many techniques that could be used to cast light into the brain structure and function. For example, it was not until the *reazione nera* was discovered by Golgi that Ramón y Cajal could carry out his seminal work in neuroscience at the turn of the nineteenth century (Ramón y Cajal and DeFelipe, 1988). Essentially, the Golgi’s staining procedure, acting over a small percentage of neurons in a brain tissue sample, constitutes some sort of a *chemical filtering procedure* which allows obtaining images of complete neurons isolated from the maze of dendrites and axons that surrounds them.

The conjunction of powerful microscopes and advanced laboratory techniques has allowed neuroscience to achieve remarkable progress in studying anatomical and morphological aspects of the brain and the elements that integrate it. Regarding brain function, though, the situation is different: at present, it is only possible to record the activity of limited numbers of cells. There are no tools that allow recording the activity of neuron circuits at microscale within large brain areas, something essential for trying to understand the most basic facts. For example, following Markram et al. (2015), “*we still lack an understanding of the cellular and synaptic mechanisms and the role of the different layers in the simplest of behaviors, such as correlated and uncorrelated single-neuron activity and, more generally, synchronous and asynchronous population activity*.” The number and variety of neuron types and morphologies, their complex interconnection patterns, the differences in synapse strength and dynamic behavior, and their changes as a result of plasticity, among many other factors, make functional analysis of brain circuits an extremely difficult task.

Detailed computational models, such as the ones presented in Wils and De Schutter (2009), Hepburn et al. (2012), Torben-Nielsen and De Schutter (2014), and Markram et al. (2015), constitute a new, promising approach for the simultaneous analysis of structure and function. Computational neuroscience methods have been already applied to the analysis of many problems during the last years, but only the availability of near-exascale computers is allowing the simulation of extensive neuronal circuits using those detailed models.

Nevertheless, the possibility of performing detailed simulations of complex neuronal circuits, interesting not only for advancing the study of the brain but also for other fields such as neurorobotics or the design of neuromorphic computing systems, requires the availability of analysis tools that are sophisticated enough for helping study what is happening in a circuit composed of tens of thousands of neurons and tens or hundreds of millions of synapses. Typical operations to be performed with these tools will be comparing simulations for discerning between normal and pathological operation, assessing the effect of certain drugs in a specific illness using simulation data, correlating interconnection patterns or morphological features with global circuit response, evaluating the influence of changing model parameters, etc. This paper presents ViSimpl, a visualization framework designed precisely for this goal which has been developed within the environment of large, multidisciplinary initiatives for brain research (Markram, 2006, 2012).

There are a number of works done to provide the visualization of simulation data with the aim to ease the analysis of the information produced. Benjaminsson et al. (2012) proposed abstract visualization of simulation data from a brain region at single-neuron level and also at neuronal population levels. They provide projections where neurons are visualized as glyphs (meaningful symbols), colored by membrane potential, while large cone-shaped glyphs are shown for spiking neurons. Also, a wireframe brain model is added to provide spatial context as well as a separated variant for the case of calcium imaging. The controls of the simulation are from VisIt software, and it also relays on ParaView, with extensions to provide *in situ* visualization. VisIt (Childs et al., 2012) is a software ready for scalar, vector, and tensor field visualization. It supports multiple mesh types, but it can be said that it is neither prepared to support particle systems nor to represent spikes or potential information.

Nowke et al. (2013) presented VisNEST to simultaneously visualize 32 vision-related brain areas of a macaque monkey. They work with simulation data that include spiking events and the mean firing rate. Selecting an area of the macaque brain allows to watch 2D simulation views. They provide information about activity, although not at a neuron level.

von Kapri et al. (2011) present a virtual reality application providing a 3D visualization of cortical layers, which reflects neuronal activity including cell membrane potential and spiking events of individual neuron (spikes are short electrical pulses by which neurons communicate). Excitatory cells are represented as pyramids and inhibitory cells as spheres.

The work by Kasiński et al. (2009) provides a 3D visualization that supports analysis of dynamical processes in spiking neural networks that include neurons, interneural connections, and spikes. It provides a set of graph windows which display the firing times or membrane potential of certain selected neurons. Sousa and Aguiar (2014) present a simulation environment that offers statistical descriptions of large networks, a builder that allows the definition and construction of large spiking network models, rich-graphical representations of the models, and their dynamical state.

In contrast with these cited papers, ViSimpl provides coordinated linked views that support the visual interactive analysis of simulation data from different perspective (structural, spatial, and temporal), at different levels of abstraction, as a way for fighting complexity, offering playback controls, focus and context clues, selecting, filtering, and sorting. These operations can be performed with individual neurons or applied to higher levels of abstraction.

The rest of the paper is organized as follows: after a short analysis of related work, ViSimpl is presented, describing its components and the way they collaborate as linked views, as well as the solutions adopted for representing spatial, temporal, and structural data. Next, results are presented and discussed, finishing with the conclusions and the discussion of future work.

## 2 Materials and Methods

**Figure 1:**
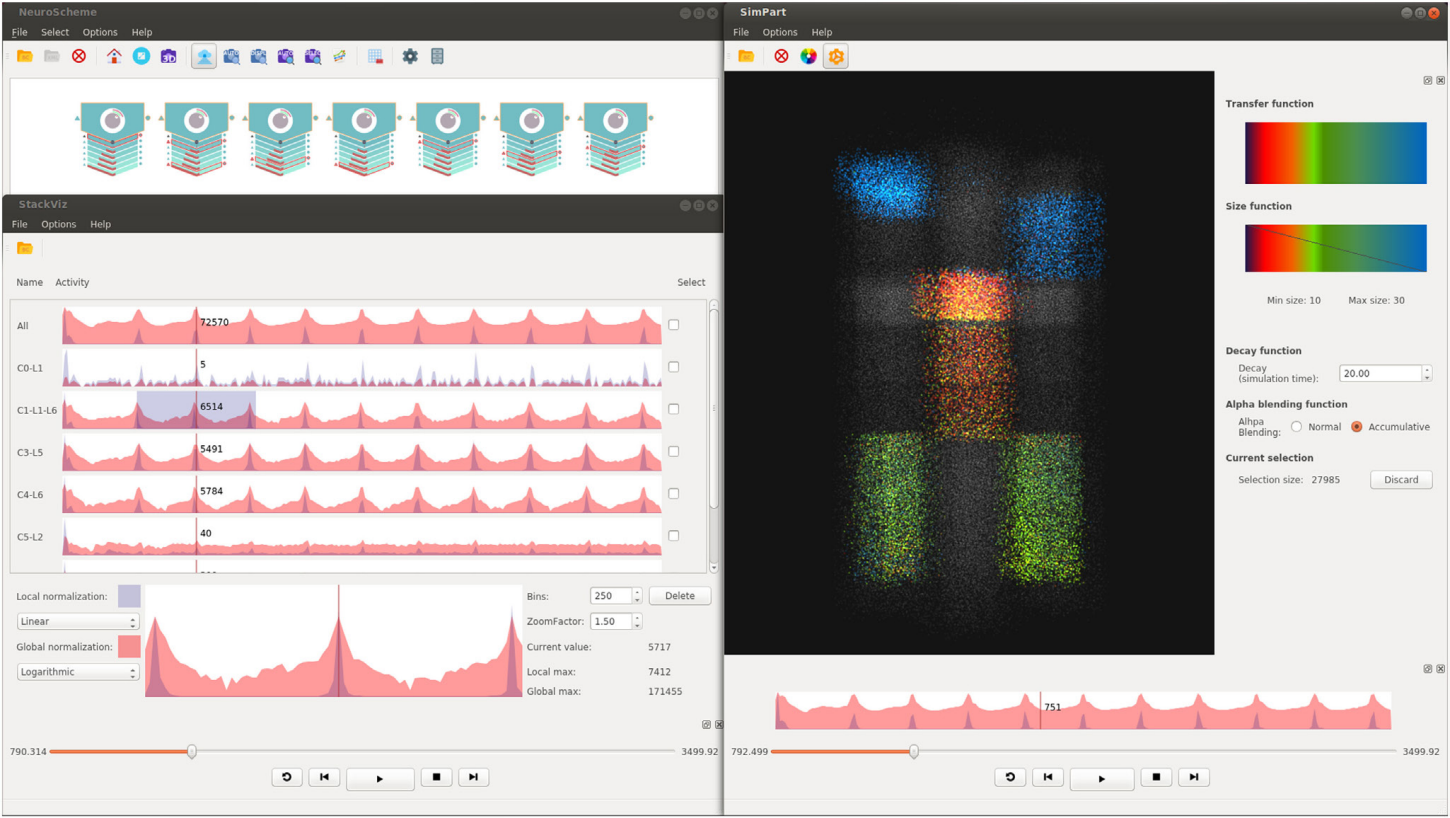
**Screenshot example of ViSimpl showing its three applications (top left, NeuroScheme; bottom left, StackViz and SimPart in the right side)**. Different layers from different minicolumns been selected using NeuroScheme, while StackViz is showing the aggregated and disaggregated graphs. SimPart is rendering the activity of the selected cells using a user-defined color-transfer function and using plain gray for the non-selected ones. This figure shows how the three views can coordinately work to enhance visual analyses by the application of selections, playback operations, play-head, and camera on the same dataset.

**Figure 2:**
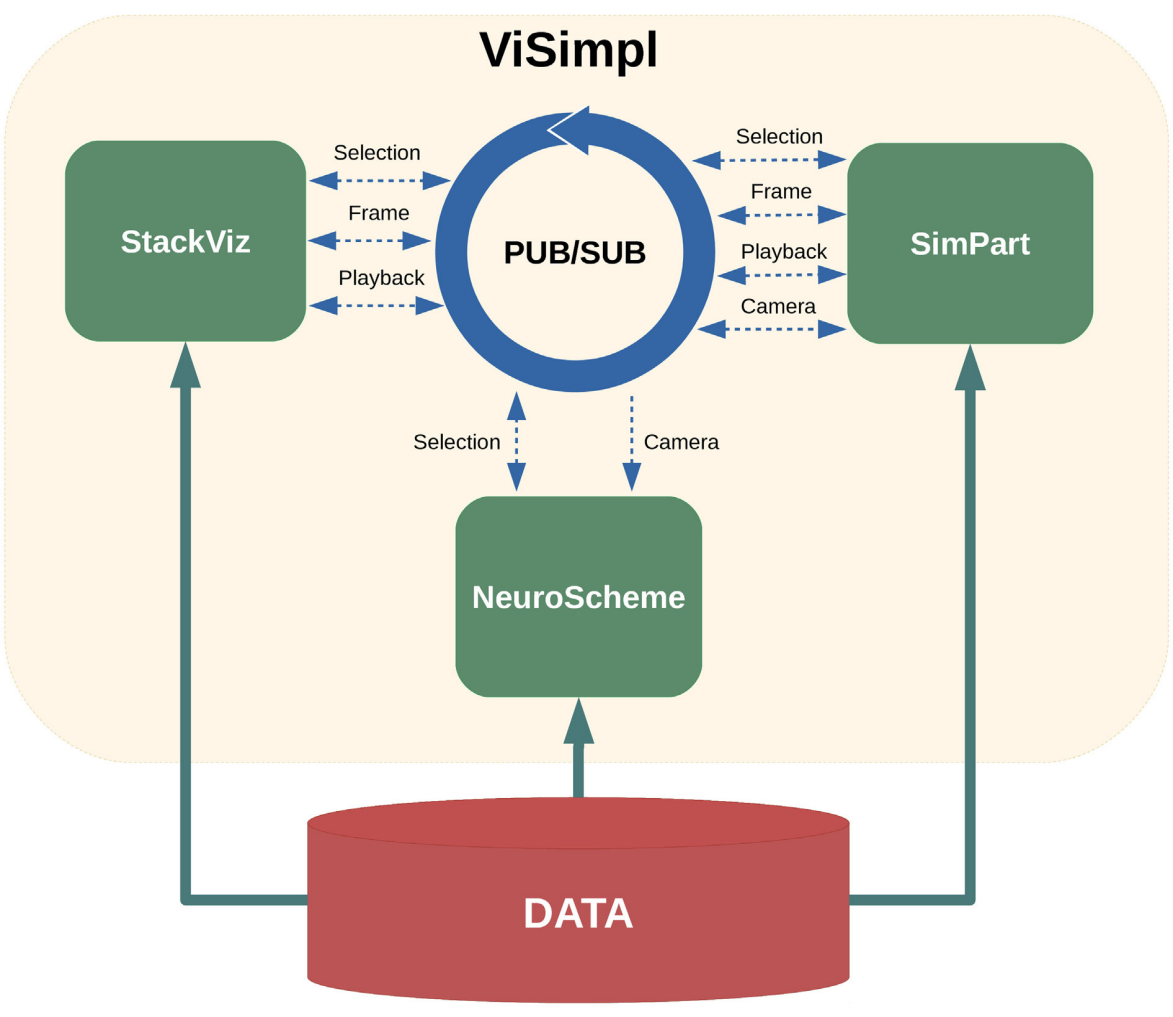
**ViSimpl general overview**. The framework relies on a publisher/subscriber (PUB/SUB) scheme that enables the communication flow among the three applications: SimPart, StackViz, and NeuroScheme. The published and subscribed topics are depicted with dashed arrows. The execution of each application can be local or distributed.

The ViSimpl framework proposed in this paper is composed of three different applications which can run in a completely independent way but which can also work together to enhance the process of navigating, analyzing, and interacting with brain simulation data and thus lead to a better understanding and improvement of the knowledge extraction. ViSimpl aims to provide visual analysis of the data from three different angles: spatial, temporal, and structural. It can be seen in Figure 1 a screenshot of ViSimpl showing its three applications, while Figure 2 depicts a general overview of the components involved in the proposed framework, showing as well how the communication among them is carried out. On one side, SimPart provides a spatio/temporal analysis of the data. On the other side, StackViz provides a temporal representation of the data at different aggregation levels. Finally, NeuroScheme allows to navigate through the underlying structure of the data using symbolic representations and different levels of abstraction.

In the following sections, the details of each of these applications and their interaction capabilities will be presented, and the linkage between views will be described.

### 2.1 Data Domain

ViSimpl visualizes one of the common types of simulation data: a set of neurons with their information from voltage and/or spikes, with the addition of information regarding their spatial layout as well as their structural and morphological information, allowing a deeper and more comprehensive analysis. In terms of temporal distribution, spikes can be considered as events, since they only have values at certain time steps, so they require to update the state of only a subset of elements over the simulation. On the other hand, voltage information has values for every time step, leading to update the state for every single element at each simulation step.

### 2.2 SimPart: Spatial Data Representation

**Figure 3:**
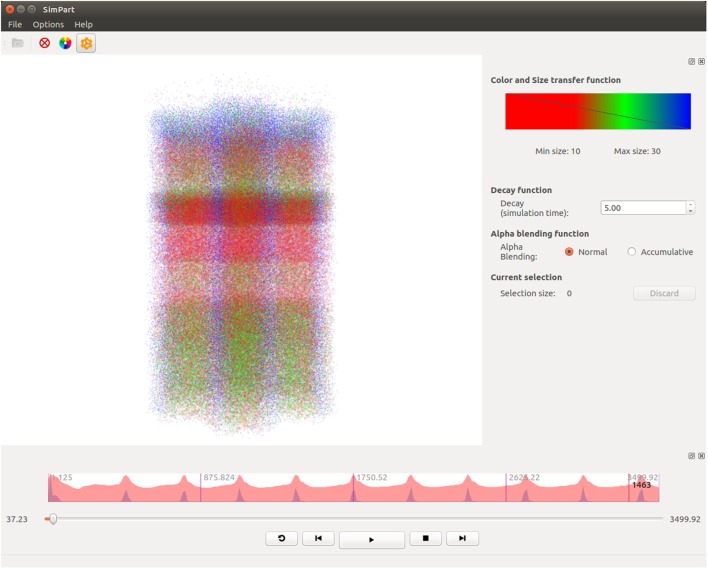
**SimPart example screenshot**. In the main widget, the particle-based activity rendering is being depicted. In the right side dock, the color-transfer function and size transfer function are placed, and below other parameters (like the decay or alpha blending type) can be tuned. In the lower dock, a summarized view of the activity is depicted on top of the playback controls.

SimPart (shown in Figure 3) is a three-dimensional visualizer for spatio-temporal data that allows to visualize brain activity simulations and to interact with data, providing the means for an extensive exploration of the large and complex datasets generated on this field. These simulations involve a certain number of neurons that interact mutually during the experiments carried out by neuroscientists exploring the connectivity and functioning of different kinds of brain regions. SimPart allows to visualize the data obtained as a result of these experiments.

In our approach, the visualization of the three-dimensional elements is performed by the use of particle rendering, a very common technique widely used on videogames and visual effects because of its capacity to visually model phenomena of diverse nature like smoke, fire, fog, and so on (Reeves, 1983), apart from their huge range of uses in other fields such as engineering and science.

Particle rendering consists in displaying a huge number of small (and computationally cheap) independent elements which could be used as a whole to create complex effects, or separately as we propose in this framework, to visualize the state of a significant number of individual elements (neurons). The use of this technique provides control of every element and capacity to alter their properties and display them all at the same time in an efficient way. The visualization of spatio-temporal data such as brain activity, usually involves thousands or even millions of elements (neurons, synapses, etc.), which fits very conveniently the common use of the particle systems.

SimPart relies on the PReFr engine (Galindo et al., 2015), a general purpose particle-rendering framework we developed for several utilities, which we have successfully used for the visualization of datasets of different nature such as GIS (Geographic Information System), neuroscientific or even point-based data, among others. This engine framework is highly configurable and provides the foundations for developing any visual effect based on particle rendering. PReFr is highly modular and is designed to allow exchanging different modules even during runtime to deploy different behaviors. For this work, its performance has been improved so PReFr is now able to render 800,000 particles simultaneously in real time on a multi-core approach. The use of this kind of traditional rendering technique comes with major advantages, as the graphics cards are designed to render geometry in the way used by the PReFr engine, since it operates under pure OpenGL. From this point of view, other techniques, such as volume rendering, might come with several drawbacks. Volume rendering is a widely used and studied technique over several environments, such as visual effects, industry, and science, among others. It is computationally intensive as it requires large amounts of process and memory in order to visualize the results. Due to its base complexity in terms of computing, it usually requires additional structures and methods to accelerate the process (Laine and Karras, 2011) that are usually generated over a preprocessing step based on the geometry and data to be visualized, but it has to be reconstructed after any modification of the involved geometry. In addition, the resolution of the grid used for this visualization is restricted to the available memory, limiting the quality of the results, and the scalability of the technique.

The described technique used on this project does not require any of these acceleration structures and is not as limited in terms of memory as volume rendering. In addition, as the render process is native to the graphics cards pipeline, there is no limitation on dynamically modifying geometry during the visualization process.

As the perceived variations in particle’s properties are mainly the color, alpha channel and their size (apart from position and other properties not used in the proposed approach, such as velocity and acceleration), SimPart exploits their possibilities to provide an effective and clear visualization. This way, color variations can be used to represent different types of elements within the same context. It can also be used to express different levels of intensities and relevance, or even to group elements.

The alpha component of the color has a special importance in this kind of visualization, as the alpha channel might quickly saturate and even impede the perception of any present elements. Therefore, since the role of the alpha component is determinant for the quality of perception, it needs to be managed carefully. The alpha component allows us to highlight elements from one another by simply decreasing their transparency and making these elements more visible than others less relevant. For example, the user can decide that the more alpha an element has, the more important the element is.

It has been included the possibility of using two types of alpha blending: the traditional one, respecting the colors set by the user, which allows to perceive the position and depth with respect to other elements. On the other hand, the accumulative alpha blending saturates the colors to create a description based on the whole set of elements behind the first ones, giving a more general perception of the elements.

In the same way, variations in size provide an effective way to represent importance, as the most important elements present on screen can be simply the biggest ones. Following this idea, the less relevant elements could be reduced to a small size that even hides them.

Both kinds of data are approached mostly in the same way, both using transfer and size function widgets to set the color and size variations, but with slight differences.

For the voltage data, colors are distributed along the whole set of possible values, mapping colors within the range established from the minimum voltage to the maximum. This kind of visualization provides a natural variation in values over time without any additional operation.

Considering the spike-based data, its direct visualization would show a set of blinking points along the whole dataset. To alleviate this problem and to provide the user a better visualization experience with this kind of data, SimPart provides a decay time widget to add a tail effect to the particles that can be configured by defining the color-transfer function over the particles life cycle. This decay allows to enhance the perception of the neurons’ state over space and time, improving the potential pattern perception conversely to the punctual or instantaneous activation, which might lack of continuity through consecutive frames. By simply varying the length of this decay, users can define the visual result ranging from blinking effects to smoother transitions. The size is managed in the same way as described for the color on both kinds of data.

The application also provides the capacity of navigating through this neuron distribution in 3D during the simulation visualization, allowing to easily localize regions of activity and to perceive patterns in the behavior of neurons. Furthermore, it can be used to explore indirect or uncertain connections between data as the varying activity leads from one region to another.

Additionally, SimPart allows to control the simulation providing the tools to manage the playback and displaying directly the desired parts of the simulation. This feature is fundamental when dealing with significantly big simulations, as the visualization of the whole set might be very long.

SimPart also provides the feature of deactivating a full region or a part of it by the use of selections, allowing the user to visualize specific subsets of elements and also to focus on a particular region, reducing the complexity of visualization and improving the knowledge extraction for the desired set or region.

### 2.3 StackViz: Temporal Data Representation

**Figure 4:**
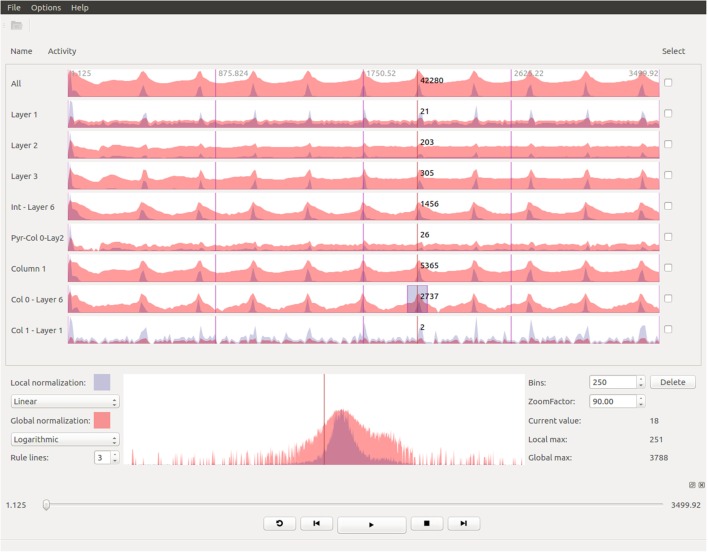
**StackViz shows, in the upper dock, an overall graph representing the magnitudes of the activity in the simulated data, on top of more graphs representing data selected using NeuroScheme**. The lower dock shows a magnified view of the region focused on the graph labeled as Col0-Layer6, on top of the simulation playback controls.

One of the key aspects of the process of analyzing brain simulation data is to understand how the electro-physiological variables evolve over time. This analysis is usually made after the simulation has been computed, sometimes with significant time lapses between the generation and the exploration of the results. In order to easily and quickly evaluate whether a simulation has been developed correctly, there is a need for tools that provide a general view of the whole dataset, even disaggregating the data into smaller chunks. Thus, our framework supports this kind of analysis and provides a representation of the data from a temporal point of view in an attempt to provide an effective and quick analysis of the obtained results. Figure 4 shows a screenshot of the StackViz tool.

The aim of StackViz is to show information about the aggregated number of spikes or the mean voltage at each time step, i.e., it shows the magnitudes of the activity of the simulation. These magnitudes are represented in the StackViz main widgets using graphs that can be stacked one below another. Therefore, when StackViz is run, it presents to the user a depiction of the aggregated activity over time, giving the users the ability to get an idea of the overall activity just at a glance and allowing them to focus their attention in the specific time step they desire.

Besides, users can select a subset (using NeuroScheme) of the data for a more detailed analysis of the activity present in that specific subset. When a subset is selected, StackViz creates a new graph below the last one, giving the users the chance to compare the activity on different subsets. One problem that arises with such comparisons is the possible great difference in the number of elements that might be present in each subset. In order to alleviate this problem, StackViz provides two graphs for each stacked widget, one using a global normalization (based on the maximum value across the whole dataset) and another using a local normalization (based on the maximum value of the subset displayed in this graph). In the case of spikes, the normalization is carried out considering the number of spikes triggered within a defined time step (depending on defined the number of histogram bins). On the other hand, voltage simulations use the mean voltage as a measure for this normalization for the current time step. The local normalized graph allows the users to get a more detailed graph both when the number of elements of the subset is small (in the case of event-based data) or when the dynamic range within the subset is small compared with the global dynamic range (in the case of dense data). This feature enables a “horizontal” analysis of a subset over time. On the other hand, the global normalization allows the users to establish “vertical” comparisons of different subsets, taking into account the graphs represent the same magnitudes on every stacked widget. The global normalization graph has been depicted in red, whereas the local one has been depicted in blue, both with some transparency. When both local and global graphs are overlapped the final color is purplish. Finally, there might be some cases where a linear representation of the activity data makes difficult to understand some of the details present in the graphs. To cope with this problem, StackViz allows the users to select linear or logarithmic scales for each of the normalization approaches.

Also, when the simulation has a large number of time steps, users might want to get a more detailed view of a specific time range. To tackle that, StackViz provides focus + context capabilities, giving a magnified view, drawn in a larger and independent widget, of a specific part of the graphs (focus) while still having the general overview of all the stacked representations (context) by simply defining a region of interest (ROI) clicking on the desired graph. The size of the ROI can be modified by dragging the edges of the region, varying simultaneously the result on the focus widget. In order to change the area being magnified, the user must have to swipe the mouse over the desired area and the magnified version of the data will automatically update. The amount of magnification can be defined by the users in StackViz’s GUI by means of a scaling factor.

Additionally, when the data to be displayed is too large, the graphs might become cluttered. In order to get a proper global view of the data, StackViz allows the user to define the number of bins in which the actual data will be displayed, providing a way to aggregate data in the temporal scale.

Taking into account all the StackViz’s features mentioned before, it can be pointed out that this tools allows not only disaggregation (in combination with NeuroScheme selection ability) regarding the elements to be analyzed (“vertical”) but also aggregations and disaggregations in the temporal analysis (“horizontal”).

### 2.4 NeuroScheme: Structural Data Representation

NeuroScheme (Pastor et al., 2015) is a tool that provides multi-scale symbolic representations and allows visual exploration of brain data from the point of view of its structure and morphology.

In this tool, abstract representations are associated with neural structures and their different morphological metrics are also displayed as visual properties of the objects. This allows to gain insight of the data in a much easier way and gives the experts the chance to understand some aspects of the data just at a glance.

Exploring neural datasets is typically challenging due to their large volume of data and visual exploration techniques can ease the process although, on the other hand, depicting such large scenarios can be counter-productive if raw data are directly visualized. To deal with this, NeuroScheme provides abstraction levels, which allow the experts to navigate and interact with them without needing to depict every object details and allowing to focus their attention on a higher level view. Besides multi-level abstraction approach, NeuroScheme also provides the ability of filtering elements based on their morphological properties, thus enhancing the exploration of large datasets.

For this work, the original version of NeuroScheme has been enhanced with filtering and sorting capabilities as well a substantial improvement of selection management, allowing a better interaction with the rest of ViSimpl’s applications.

**Figure 5:**
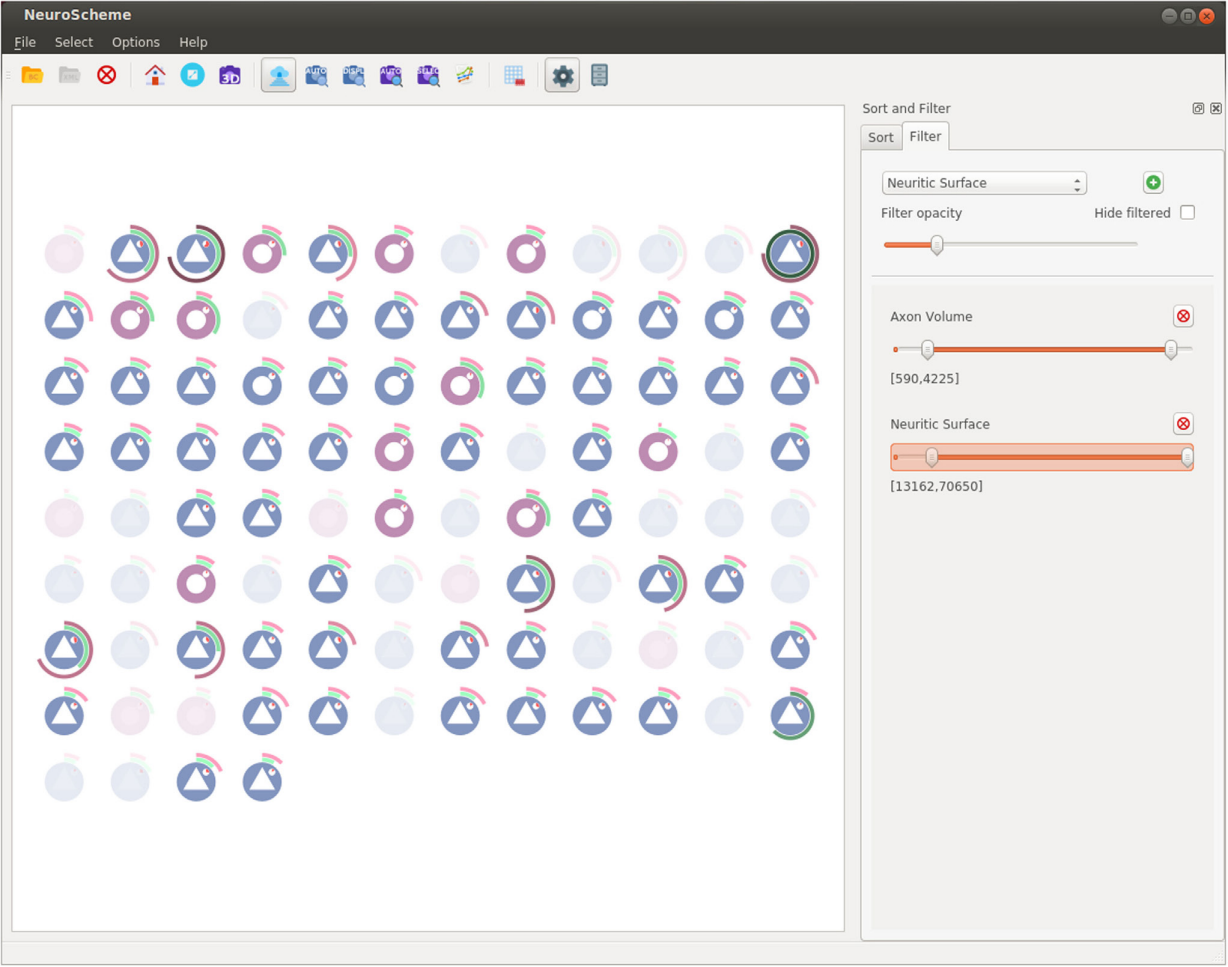
**NeuroScheme at neuron level using its grid layout**. Icons represent different morphological properties of the cells. In this screenshot, a filter has been set, and the faded icons represent the neurons being filtered out.

Figure 5 shows a capture of NeuroScheme at neuron level. In this example, each of the depicted symbols represent a specific neuron and its color, icon, and visual properties represent, for example, the morphological type of neuron or its physiological type or other morphometric properties (surface, volume, dendritic bifurcations, etc.). These mappings can be user defined but for the presented example the color has been mapped to the physiological type (reddish for excitatory cells, bluish for inhibitory cells); the symbol in the center of the glyphs represents the morphological type (triangle for pyramidal cells and circle for interneurons cells); the pie chart besides the symbol represents the number of dendritic bifurcations; the rings represent volume and surface of both soma (inner ring) and dendrites (outer ring), using in each case the brightness of color (greenish for soma and reddish for dendrites) for volume and the angle for surface. In the presented example, some neurons have been filtered out using “Axon Volume” and “Neuritic Surface” ranges. In this filtered view, opacity is being used to mark the neurons being filtered out, but NeuroScheme also supports filtering by hiding elements and rearranging the grid and sizes of elements in order to get more space for the remaining elements. Finally, Figure 1 shows NeuroScheme working as part of the proposed framework. In this case, the view is depicting the cortical column level of abstraction, which shows the number of pyramidal and interneuron cells per layer as well as a representation of the average cell. Selection at this level can be performed in several ways (by morphological type of neuron, by layer, by type and layer, etc.) and are marked using a red or orange border indicating fully or partially selected entities, respectively. A detailed explanation of the glyphs and their meaning can be found in Pastor et al. (2015).

### 2.5 Linked Views and Interaction

As stated before, ViSimp is composed of independent applications which can boost their analysis capacities when working together. To achieve this, our framework uses a publisher/subscriber (PUB/SUB) scheme relying on the ZeroEQ open-source project (ZeroEQ, 2016), developed in the context of the Human Brain Project. ZeroEQ implements Zeroconf (Steinberg and Cheshire, 2005) protocol allowing automatic discovery and enabling late join connections. This means that any of the ViSimp applications can be launched at any time or can be executed more than once or even run in different computers. This decoupled model has great benefits. First, all the applications can work as linked views as if they all were a greater and unique application but, second, as it allows the users to run each application on different computers can lead to several interesting configurations. For example, SimPart, which needs more 3D rendering computing power, could be run on a high-fidelity display infrastructure while the users can be running all other applications on their laptop or hand-held devices, such as advanced smartphones or tablets. Another example would be when users want to collaboratively analyze data. In this case, they could run the applications in different locations and interact with the data while getting the results of remote user interaction. This approach has also the advantage of being extensible by adding additional applications that can act as new views which perform other type of analysis and link them to the existing ones with a small integration cost.

Figure 2 summarizes this PUB/SUB approach and the type of messages each application is publishing or subscribing to. In the following sections, the details of these messages will be described.

#### 2.5.1 Selections

One feature that can help getting a better understanding of large and complex data is the ability to focus on subsets or regions of interest. In the proposed approach, this is supported in different ways. One of them is being able to visually select a subset of neurons using NeuroScheme application. NeuroScheme allows not only to select individual cells but also to perform selections at different scales based on morphological metrics and properties. Also, some of these levels also allow to select elements using other inherent properties (type of cell, layer, etc.). NeuroScheme also supports filtering neurons using many morphological properties, which allows again to focus on some cells and to select them easily both *via* rubber-band selection or just selecting all the visualized cells. Additionally, NeuroScheme also supports sorting and filtering operations that enhance the data exploration and thus, the extraction of knowledge. Furthermore, the combination of these sorting operations with rubber-band selections provides an easy way of defining subsets. Finally, rubber-band selection can be very useful combined with NeuroScheme’s 3D view to focus on a specific spatial region. All the selections made can be stored and named for an easy recall of the selection state.

Once a selection has been established in NeuroScheme, it can be published so both StackViz and SimPart can subscribe to the selection topic allowing the users to focus their attention on a specific subset of cells and thus get a better understanding of their activity. On one hand, StackViz will show the temporal activity allowing to easily compare subsets of the data. On the other hand, SimPart will spatially render the activity of the selected cells while the rest of the cells are rendered in semi-transparent gray at the user’s convenience, providing in that way a context of the whole dataset.

The selection PUB/SUB scheme used in ViSimpl framework can be seen in Figure 2.

#### 2.5.2 Playback

As stated before, both SimPart and StackViz can act as controllers of the simulation playback state. It means that both of them can not only publish playback events but also subscribe to these events. One of the benefits of outsourcing the playback control from the actual renderer is, e.g., that users can control the simulation playback from their laptop or hand-held while it is being rendered in a high-fidelity display infrastructure.

The playback events are the usual ones: play, pause, stop, go to beginning, enable loop, etc. Also, an event for establishing the current play-head frame has also been implemented (“Frame” event as depicted in Figure 2). This event allows to set the play-head both from SimPart and StackViz. In the latter, it is also possible to click on any of the stacked graphs and move the current play-head to that specific point. This feature allows users to easily jump to the time steps where, based on the information being visualized in StackViz, they evaluate whether relevant activity is present. Playback can also be controlled from SimPart, allowing this application to be self-sufficient to perform spatial analysis of the simulation data.

#### 2.5.3 Camera

NeuroScheme application has two layout modes for displaying sets of elements. On one hand, it provides a grid layout, which is useful, for example, to perform sorting and filtering operations combined with rubber-band selections. On the other hand, it also provides a 3D-based spatial layout. This kind of layout uses 3D camera parameters for computing the 2D positions of the elements mimicking the positions of the elements rendered spatially, as for example is done in SimPart. This way SimPart publishes its camera view matrix and NeuroScheme subscribes to that topic, thus both applications have their 3D-view synchronized. This mode allows side-by-side analysis and comparison between different view modalities such as, in our case, NeuroScheme and SimPart views, as Figure 8 shows.

## 3 Results and Discussion

ViSimpl is a completely portable software, tested under OS X, Linux, and MS Windows (7, 8.1, and 10), and programed using C++, Qt 5.4, and OpenGL 3.3. The proposed framework has been tested with different datasets; the one that appears in the Figures of this paper was provided by The Blue Brain Project (Markram, 2006) and contains 217,000 neurons.

This dataset provides simulations carried out over a cortical circuit and thus, it presents a *vertical* organization of the neuron population into 7 columns with 310 minicolumns each. The neuron population can also be decomposed *transversely* or *horizontally* into six layers, labeled I to VI (from the outermost to the innermost layers in the brain). Besides, neurons’ morphologies are also available in this dataset, allowing NeuroScheme to provide a visual representation for them and use morphometrics for filtering and sorting operations.

Specifically, regarding simulation reports, the dataset contains voltage magnitudes and spikes (approximately 2.5M), which are Boolean-valued impulses distributed sparsely in time.

To access this dataset, the open-source Brion library has been used (Brion, 2016).

**Figure 6:**
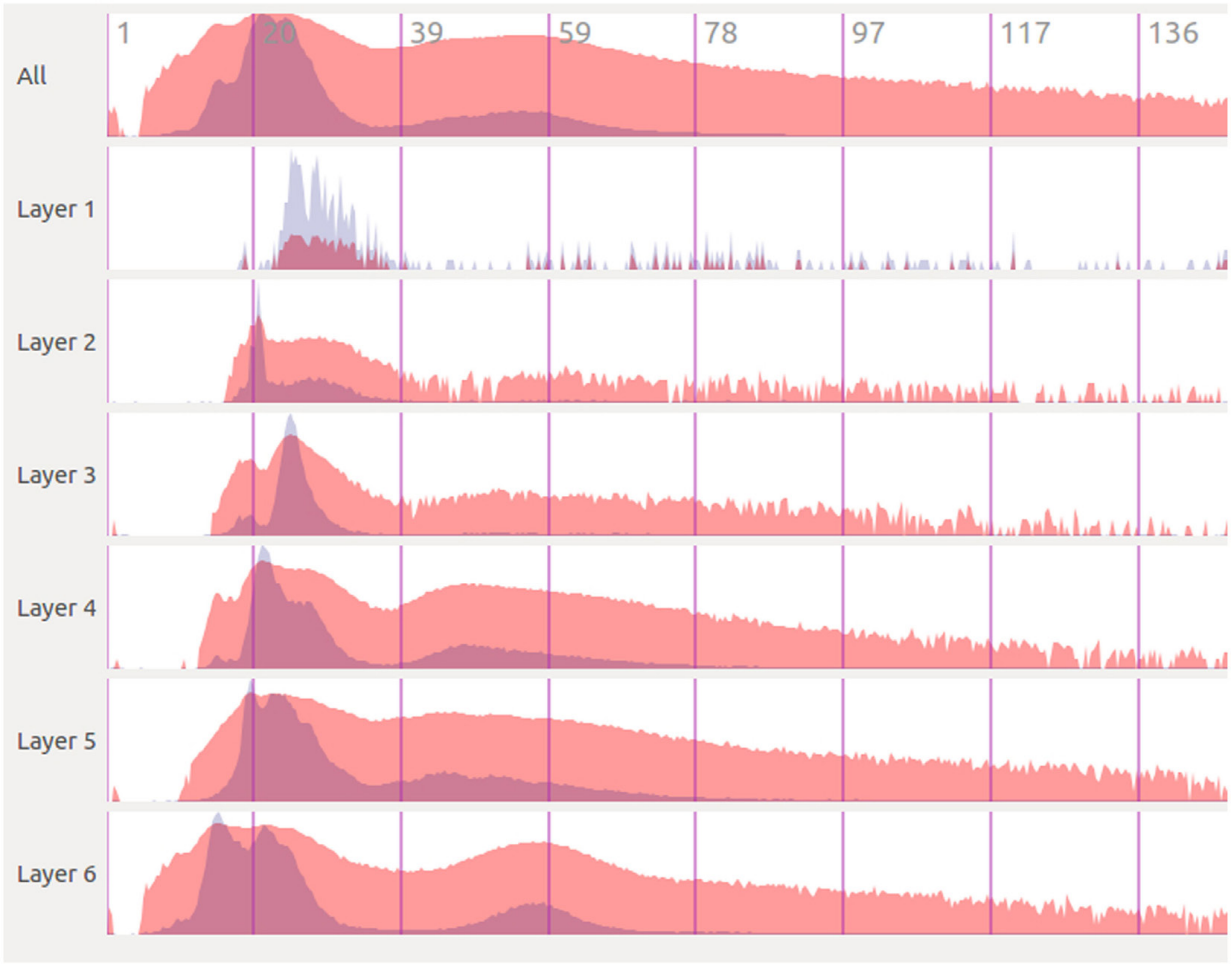
**Extraction of temporal analysis views of a region with StackViz**. This region corresponds to the lapse from 1.125 to around 150 in simulation time. The first row represents the aggregation of the whole dataset, while the rest are representations of subsets corresponding with to different layers. Global normalization is depicted in red, while local normalization is depicted in blue. This view allows to do comparisons both over time (“horizontally”) and among subsets (“vertically”).

Figure 6 shows an example of the kind of temporal analysis that can be performed with StackViz. In this figure, all of the graphs show information corresponding to a small region where the stimuli defined in the simulation induce a specific response. The first row (labeled *All*) represents the aggregated information of the whole dataset, while the following rows show partial information, gathered in this case at specific layers, allowing the computation of disaggregated graphs in order to perform in-depth analysis operations. Additionally, the graphs show local normalization with linear mapping in a bluish tones and global normalization with logarithmic mapping in reddish tones. The areas where local and global normalization are superposed are represented in purple. It is important to note that the set of neurons included in each particular selection can be picked by performing selections using NeuroScheme at different levels of abstraction, simplifying therefore user operation. Providing top-down selection mechanisms is essential to deal with complexity in ever-growing datasets; in this example, belonging to a specific layer has been used as the property to disaggregate, but other criteria could have also be used.

The plots in Figure 6 contain plenty of information that can contribute to a deep comprehension of the events that take place within a simulation. In this example, it can be analyzed at a glance how the activity in the simulation occurs among layers. It can be easily seen in this figure that neuron activity starts first in layer 6, being a quite small impulse regarding the size of the dataset, that can only be seen in this plot with global normalization (due to its logarithmic mapping). Just a few simulation steps afterward, the activity increases dramatically in layer 6, propagating later on across the other layers. The local normalization graphs allow also extracting valuable information. It can be seen, for example, that layer 6 starts with a large peak (which is actually composed of two peaks), goes afterward through a period of inactivity, and finishes generating another peak, this time smaller. This evolution is not that evident if only globally normalized information is visualized. Additionally, the global normalization allows to extract information “vertically,” letting the user compare each graph. For example, the amount of activity each layer has at each time step can be nicely compared, and also the intensity of their peaks are one compared to another.

**Figure 7:**
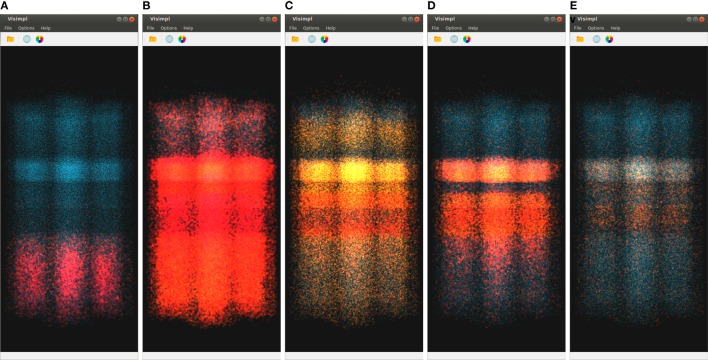
**SimPart’s spatio-temporal visualization of the test data at different time steps (labeled below each subfigure)**. The color-transfer function goes from red to blue with orange in between. SimPart allows the analysis of the spatial propagation of the activity along the cells over time, where different patterns emerge through visual aggregation. **(A)** 15.23, **(B)** 20.13, **(C)** 42.47, **(D)** 55.31, and **(E)** 84.47.

Figure 7 shows five captures of SimPart, each of them rendering the network activity for a specific time step. Again, these time steps are quite close in time, showing the activity induced by the same original impulse analyzed in Figure 6. The color-transfer function chosen for this example goes from red (activation) to blue (rest). It can be seen that in the first time step (Figure 7A) most of the cells remain in a rest state, although a number of cells on the lower part of the image (corresponding to the inner cortex area, layer 6) are beginning to spike. This activity spreads vertically over time, being possible to notice that activity starts in layer 6, spreads later on to lower layers, and ends finally in layers 1 and 3.

In a way, it can be said that these two figures complement each other. Precise timing of neuron firing per layer can be measured with much more accuracy in Figure 6, but Figure 7 provides richer spatial information. Furthermore, although previous discussion can be performed with either SimPart or StackViz working independently, the information provided to the user is greatly leveraged when both applications work as linked views and rely on NeuroScheme’s exploratory and selection capabilities. This way, the play-head moves synchronized in both views allowing users to get a better understanding of the activity in a specific simulation step, having the whole temporal context in StackViz and the whole spatial context in SimPart.

Also, the application design allows users to jump to a specific simulation time step by clicking on one of the StackViz’s graphs, which in this case would allow users to move the play-head, for example, to one of the peaks that they want to analyze in detail. Putting NeuroScheme in the loop as the third view of the data also facilitates navigating, finding, and selecting the structures which the user wants to place the focus on. In the example shown in Figures 6 and 7, the user has focused the analysis toward cortical layers by selecting them through NeuroScheme. This selection is reflected then in the way data are disaggregated in order to perform deeper analysis.

**Figure 8:**
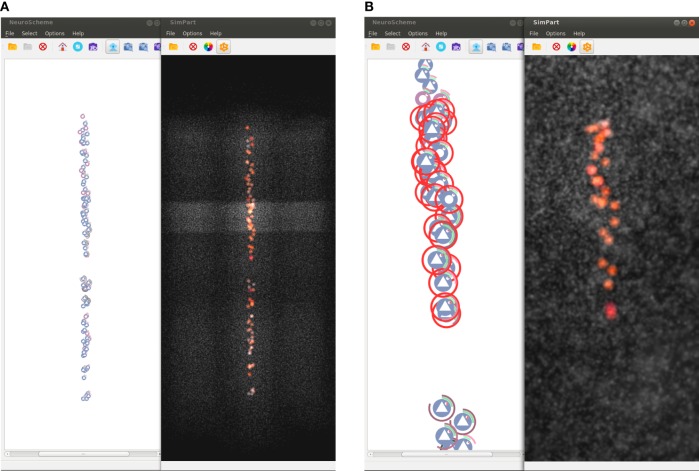
**Screenshots of selection examples using NeuroScheme’s 3D mode**. These figures show how both spatial particle-based views can be coordinately used with NeuroScheme’s abstract representations for analyzing subsets of the data. Non-selected cells are shown in gray in order to get a spatial context representation of the whole subset. **(A)** Selection of all cells displayed in NeuroScheme. In this case, the selection contains the cells of a specific minicolumn. **(B)** Rubber-band selection carried out on NeuroScheme and SimPart focused on the specific selected ROI.

Figure 8 shows another possible configuration of this framework, in which NeuroScheme and SimPart work as linked views. In this case, NeuroScheme has been configured to display neurons using a 3D-based spatial layout, for using levels of abstraction to facilitate the selection of a specific minicolumn (Figure 8A). After this minicolumn is chosen, the user can perform a rubber-band selection, in order to focus on a specific ROI (Figure 8B). This 3D-based spatial layout can be helpful for finding patterns in the abstract representation that can be related to events occurring in the simulation, or the other way around. By contrast, Figure 1 shows a selection of different layers from different columns in NeuroScheme, this time in grid mode. This selection is also shown in SimPart while playing the simulation, as well as information from the layers is also displayed in StackViz. All these different options in data selection and display result in a very rich visualization and interaction process that allows users to analyze the data in a quite exhaustive way, facilitate searching for patterns and structures in the data, and provide new insight about the simulations and how it relates to circuit structure or morphology.

Another feature that comes up with the use of alpha blending and billboards for visualization purposes is the potential emergence of patterns due to visual aggregation, for example, by the accumulation of colors that derives in the appearance of in-between colors as a result of the render blending process. This can be appreciated in Figure 7 as new colors like the purplish present in Figures 7A,D, the whitish red appearing in Figure 7B, the greenish in picture Figure 7C (top and bottom of activity regions), and the whitish in the Figure 7E picture on the middle band. These effects provide a summarized vision of the activity undergoing on these regions after zoom out operations, letting users perceive that the global activity within that region is a mixture of several combined activity states. This feature can be very useful in order to understand the global state of dense populated regions by simply playing with the alpha component of the transfer function and the blending mode.

Summarizing, it can be stated that ViSimpl allows neuroscientists to interactively visualize the temporal, spatial, and structural distribution of the simulation activity of large neuron populations, giving them at the same time the ability of selecting which of the neuronal network areas will be displayed and grouped, how neurons will be aggregated and how spatial and temporal information will be displayed. This is particularly important as the complexity of the circuits being simulated increases, in order to allow users get full advantage of the simulation results.

## 4 Conclusion and Future Work

This paper presents ViSimpl, a framework that helps users to visually analyze complex neuron-level detailed brain simulations. ViSimpl allows users to perform simultaneous temporal, spatial, and structural selection operations, in order to facilitate the analysis of complex datasets such as those produced in large scale simulations.

ViSimpl is composed of three different applications: the first one, SimPart, provides the spatio-temporal analysis capabilities based on a 3D particle-based rendering approach. The second one, StackViz, is the application that provides the temporal information associated to the simulation data, supporting different data aggregation and disaggregation operations while giving simultaneously focus and context clues. Finally, NeuroScheme, the third application, provides a multi-scale symbolic representation which allows performing selection and filtering operations in order to facilitate user navigation along neuroscience structured data, which is specially important to deal with the degree of complexity found in large scale simulations. The most interesting feature of ViSimpl is that the three described applications can be seen as linked views, acting in a completely coordinated and synchronized way. In consequence, ViSimpl provides users with a powerful and complete tool for interacting with the simulation, and most important, for enhancing the process of knowledge extraction from the simulation results.

Preliminary work with ViSimpl has shown that it is a flexible tool that can help during the analysis process of the results of large, complex simulations such as the ones that are being carried out within the HBP project. Nevertheless, there are a number of issues where future work is needed:

- First, each application integrating ViSimpl can be run on an independent machine, thus a specific machine’s computing power does not have to be shared among all of the applications, and more important, different machines can be selected for each application, depending on their specific features. Regarding computational aspects, there is a problem nowadays with the particle-rendering stage, when the number of elements to render is very large: at the moment, there are many steps that are being carried out in the CPU, which affects performance; it has been found that the number of particles that can be rendered on an i7-based desktop computer should not be too large (around 800,000). It is planned to re-implement the particle-rendering pipeline in GPU, which should boost the number of particles up to millions of them.
- Regarding SimPart, a predefined set of well-defined color and size transfer functions will facilitate the user experience, in particular for new users. Also, letting users define, save, and recall custom transfer functions will facilitate the simulation analysis task.
- Also regarding SimPart, a more sophisticated focus and context approach will be an additional improvement for users who want to study a specific set of cells. The exploitation of other particles’ features, such as speed and acceleration, will be explored in the near future for improving the transference of information.
- At last, another aspect to be explored is the type of graph generated by StackViz; there are many methods that can produce more compact and understandable graphs, such as Horizon Charts or StreamGraphs.

## Author Contributions

All authors conceived of the project. SG, PT, and OR worked on the design of the system. SG and PT worked on the implementation. All authors reviewed and contributed and approved the final version of the manuscript.

## Conflict of Interest Statement

The authors declare that the research was conducted in the absence of any commercial or financial relationships that could be construed as a potential conflict of interest.

## Supplementary Material

Video 1 **A demo video is provided for this paper**. Further details can be found at ViSimpl website.
